# Expansion, Exploitation and Extinction: Niche Construction in Ephemeral Landscapes

**DOI:** 10.1038/s41598-020-66888-9

**Published:** 2020-06-22

**Authors:** Miles T. Wetherington, Juan E. Keymer

**Affiliations:** 1 Department of Ecology, School of Biological Sciences, P. Catholic University of Chile, Santiago, Chile; 2 Biological Research Centre, Institute of Biophysics, Szeged, Hungary; 3 Institute of Physics, School of Physics, P. Catholic University of Chile, Santiago, Chile; 4 Department of Natural Sciences and Technology, University of Aysén, Coyhaique, Chile

**Keywords:** Community ecology, Ecological modelling, Evolutionary ecology, Population dynamics, Theoretical ecology, Phase transitions and critical phenomena

## Abstract

We aim to understand general consequences of niche construction on metapopulation dynamics in ephemeral landscapes. To this effect, a contact process-like stochastic spatial model is introduced where local populations colonize and go extinct on a dynamic landscape of habitable and destroyed patches. In contrast to previous models, where the extinction threshold is a consequence of available niche rendered by global rates of patch destruction/renewal, here we investigate how the metapopulation persists when they are the sole generators of their own niche. Niche construction is full-filled by localized populations through the transformation of destroyed patches in their neighborhood to viable habitat for future colonization. With this theoretical framework we are able to address the dual nature of niche construction by investigating the ephemerality of the landscape (destruction rate) and the continuum of population level strategies, where construction comes at a cost to colonization. Using mean field theory and Monte Carlo simulations of the model, we are able to quantify optimal population level strategies in a wide range of ephemeral landscapes. Interestingly, we observe qualitative differences at the extinction threshold between analytic and numeric results. Investigating this discrepancy further, we find that increasing niche construction neighborhood in the spatial model leads to two interrelated effects *i*) an increased rate in range expansion *ii*) a loss in resiliency and return of the discontinuous transition at the extinction threshold. Furthermore, in the discontinuous regime of the model, spatial clustering prior to a critical transition disappears. This is a significant finding as spatial clustering has been considered to be an early warning signal before ecosystems reach their ‘tipping point’. In addition to maintaining stability, we find local niche construction strategies have an advantage when in scramble competition with an exploiter strategy because of their ability to monopolize the constructed niche due to spatial adjacency. As the niche construction neighborhood expands this advantage disappears and the exploiter strategy out-competes the niche constructor. In some cases the exploiter pushes the niche constructor to extinction, thus a tragedy of the commons ensues leading to ‘ecological suicide’ and a collapse of the niche.

## Introduction

Understanding the co-regulatory feedback between living systems and their environment is a primary goal driving ecological research^1–3^. Over the past quarter-century, research has primarily focused on studying the differences between ecological and evolutionary dynamics^3,4^, most notable of these are the closely related concepts, ecosystem engineering and niche construction. Ecosystem engineering occurs within the lifetime of an individual in a population, and is defined as its modification of the microenvironment within which it makes a living. In turn, the consequences of these actions have an impact on the coupled physical environment and ecological community (i.e. the ecotope^5^). Ecosystem engineers play an important role in the restoration and maintenance of the landscape^6^, the resilience of ecosystems^7^ and are often regarded as keystone species due to their role in stabilizing communities^4,8^.

An ecological interpretation of niche construction includes the mechanisms of ecosystem engineering along with all byproducts comprising the life-history of the organism (behavior, nutrient uptake, and excretion for example detritus^9^, etc.) which may be considered more indirect forms of modification^10^.

The microbial world is typified by such feedback processes; for example, the exchange of metabolic byproducts plays an important role in creating and maintaining interdependent or ‘syntrophic’ relationships where two or more microbial groups live symbiotically via nutrient cross-feeding^11^ or the transaction of other resources^12^. Such microbial consortia act as their own ecological units, often coexisting in harmony^13^ and expanding beyond the niche of any one strategy. Furthermore, such niche construction has a tighter feedback to the ecological unitpopulation in question and can play an important role in influencing their evolutionary trajectory even playing a vital role in biogeochemical cycles^14^. In this way niche construction is a life history trait which has the potential to expand or maintain the ecological a populations niche, including the trans-generational inheritance of improved local conditions (ecological inheritance). Alternatively, once this strategy emergesin the biosphere, it becomes vulnerable to exploitation, either from within the population or nearby strategies in competition for similar resources. If exploitation does not eliminate niche construction and this new exploiter-victim interaction becomes tightly coupled, exploiters will continue to profit by expanding into otherwise inaccessible regions^15^.

The landmark work of Krakauer *et al*. addressed ecological and evolutionary consequences of niche construction in a Lotka-Voterra competition framework^16^. By shifting the focus from population genetics to an emphasis on ecologicalpopulation dynamics and the mechanisms for niche control, this approach differed from earlier theoretical investigations^17,18^. We will briefly describe their system and summarize some of their findings as their work has inspired the model considered in this investigation. They introduce the constructed niche directly as an additional state variable in the dynamical system. If the niche is invested in by the developing population it acts as a public good, increasing the potential carrying capacity of the population. In this way the abiotic environment feeds back into the ecologicalpopulation dynamics influencing the state-space over which a single strategy the population can persist in solitude i.e. its fundamental niche. There is a trade off to investing in the niche, notably, by allocating resources (energy/time/proportion of the local population) towards its construction and maintenance ($c$), one is inherently appropriating them away from immediate colonization of territory or resources ($1-c$). In the case where the niche must be constantly invested in, due to external perturbations or dissipation for example, a balance between colonization and construction must be found.

This dilemma can be resolved via specialization within the population giving rise to a division of labor. Classic examples being the specialization of tasks between germ-line and somatic cells^19,20^ or the caste system in insect societies^21^. Alternatively, the costs of construction can be spread throughout the population evenly. This is common in microbial systems, for example in budding yeast *Saccharomyces cerevisiae* which, in order to metabolize sucrose, secretes a digestive enzyme (invertase) into the environment^22^. A similar mechanism has been well documented with *Pseudomonas aeruginosa* which, in iron-limited conditions, will begin to excrete an ‘iron scavenging’ extracellular compound (siderophore) to bind and transport iron across the cell membrane^23^.

In single strategy ecosystems like these aforementioned microbial systemsits simplest form (logistic growth of a single strategy) a realization of the niche is maximized bypopulation strategies which allocate resources evenly between niche construction and reproduction (where $c\approx 0.5$). However, when placed in competition with other strategies the niche acts as a common-pool resource, in other words it is nonexcludable and limited. Therefore, if 2 strategies compete and extract from this common-pool resource, the one with the lowest allocation towards niche construction (the exploiter strategy) dominates, eventually pushing the other to extinction^24^. This has been shown to be the case explicitly in well-mixed versions of the aforementioned microbial systems; when wild-type strategies are in resource competition with exploiter strategies that do not excrete the costly enzymes/compounds they go extinct^25,26^.

Notice, however, in driving the other strategy to extinction the exploiter loses access to the portion of the niche that was constructed, which was also verified experimentally (See Fig. 3C in^25^). Logically this leaves us with the dilemma that evolutionary and ecological dynamics should push niche construction as a strategy to extinction. Herein lies the tragedy of the commons, where the resource in question has been manifested by niche construction^27^. In an environment where habitat renewal is not free and the fundamental niche is not inherent to the environment, for example cases where disturbances are frequent^28^, local facilitation is necessary for establishment (see^29^ for a review) or the ecosystem is susceptible to critical transitions^30^, this scenario can lead to a form of evolutionary suicide (^31^ and see^32^ for a review).

With this dilemma laid out before us, and with these microbial systems in mind, how is niche construction maintained?

One universal yet often overlooked feature of niche construction is its relative spatial structure and scale compared to other ecological interactions at play, for example colonization. For laboratory settings of the microbial systems mentioned, well-mixed conditions ensure that niche construction is global. Whereas outside these simplified laboratory scenarios, real populations are spatially distributed generating a population of populations, i.e. metapopulation^33^. In this natural scenario the scale of niche construction is limited by the rate of diffusion^34^, i.e. it is relatively localized to subpopulations within the metapopulation. Therefore, accessibility of the constructed niche can range from a completely privatized good to a common pool resource. Studying this transition within the excludability spectrum from monopolized to exploited is the goal of this work. Since it is a broad concept, we narrow our view of niche construction to the mechanism by which an ecological unit (population, microbial consortia, etc.) renews the necessary and sufficient conditions for persistence in its surrounding environment. Opposing this effort is the dissipation of order from the external environment, for example disturbances observed in ecosystems which are a characteristic found at all scales of biological organization (meteorite impacts, forest fires, antibiotic injections etc.).

**Figure 1:**
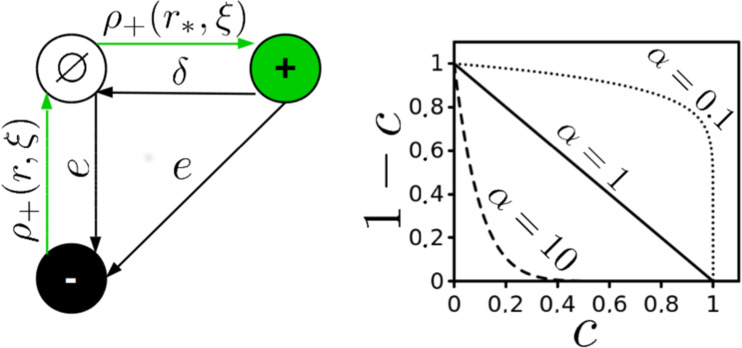
NC Reactions and the Colonization-Construction Trade-off. Left: Schematic showing the reactions for the NC model. The probability of colonization $(1-c)$ and construction *c* events occurring are dependent on the density $(\rho )$ of occupied sites within the colonization (${r}_{\ast }$) and construction range ($r$) of the stochastic process $(\xi )$. Sites in the lattice acquire one of 3 possible states, $S=\{-,\varnothing ,+\}$ so the state of the system at time $t$ is ${\xi }_{t}:{\mathscr{L}}\to S$. Right: Trade-off between construction (x-axis) and colonization (y-axis) with respect to 3 different values of *α*.

In the next section we introduce the contact process, a general model of spatial colonization and extinction dynamics. Giving some background, we then develop our niche construction model. For those not interested in the specific mathematical details of the model, the main features can be found in Fig. 1 with the models Markov diagram and the colonization-construction trade-off.

Equipped with this model, we ask the following questions; how does the spatial structure of niche construction effect *i)* metapopulation resilience and qualitative behavior at the extinction threshold in the face of disturbances, *ii)* spatial range expansion at the scale of the metapopulation and, *iii)* the overall carrying capacity with respect to disturbance, and finally, *iv)* when in direct competition with an exploiter strategy, in what scenarios should we expect to see constructor dominance? exploiter dominance? and coexistence?

## The Model

### The contact process

As a point of departure for our model, we begin by introducing the contact process (CP)^35^, an interacting particle system^36^ which follows colonization and extinction dynamics of particles on a countable set of spatial locations (${\mathcal L}$). For our landscape we will consider a 2D lattice consisting of 256 by 256 sites with periodic boundary conditions ${\mathcal L} ={{\mathbb{Z}}}_{256}\times {{\mathbb{Z}}}_{256}\cong {{\mathbb{Z}}}_{256}^{2}$. On the landscape ${\mathcal L}$ populations (occupied patches) go extinct (at rate $\delta$) and recruit adjacent habitable patches (at rate 1) stochastically and in continuous time. When we say some event happens at rate $q$ this signifies that given parameter $q$ the time between occurrences has an exponential distribution $P({t}_{i}\le t)=1-exp(\,-\,qt)$ and a mean of $\mathrm{1/}q$. Patches in the landscape $(x,y\in  {\mathcal L} )$ can be in one of two states $S=\{\varnothing \mathrm{,1}\}$ which correspond to vacant habitat and occupied habitat, respectively. Consequently, we can describe the state of site $x$ at time $t$ as ${\xi }_{t}(x)$. Hence, ${\xi }_{t}$ assigns a state $S$ to all sites in the landscape: ${\xi }_{t}: {\mathcal L} \to S$. Considering the colonization and extinction dynamics of individual patches, we are left with the defining reactions of the contact process:

$$\begin{array}{lll}{1}_{x} & \mathop{\longrightarrow }\limits^{\delta } & {\varnothing }_{x}\\ {\varnothing }_{x}+{1}_{y} & \mathop{\longrightarrow }\limits^{1/{Z}_{\ast }} & {1}_{x}+{1}_{y},\end{array}$$

where the colonization neighborhood Z_*_ for site $x$ is defined by the interaction range r_*_ using the Hamming coordinate system:

$${Z}_{\ast }(x,{r}_{\ast })=\{y\in  {\mathcal L} :\parallel x-y\parallel \le {r}_{\ast }\}$$

As an example, if ${r}_{\ast }=1$, each patch has four neighbors (North, East, South and West). In this scenario, if our focal patch is vacant we pick a neighbor randomly, if the neighbor is occupied it colonizes with probability 1. Thus colonization dynamics are dependent on the conditional probability of neighboring patches being occupied, which in turn are dependent on their neighbors, and so on. Following this it is easy to understand that an exact analytic solution to the CP is not feasible due to its spatial structure. That being said, several important features of the model have been proven rigorously and its significance in the study of absorbing state phase transitions is well documented^37^. A first attempt to understanding the dynamics of the model requires an investigation into the mean field approximation (MFA). This approach assumes the system to be ‘well-mixed’, in other words, the spatial structure is disregarded and the effect of all other sites ($p$) is approximated and by an averaged effect, thus reducing the many-bodied problem to a one-bodied problem:

1 $$\dot{p}=p(1-p)-\delta p$$

Incidentally, this also happens to be Levins equation of metapopulation dynamics^33^. Setting $\dot{p}=0$ and solving for $p$ we arrive at the steady state equilibrium:

2 $$\widehat{p}=1-{ {\mathcal R} }_{0}^{-1}$$

We have introduced a new term, the ‘basic reproductive number’, ${ {\mathcal R} }_{0}=1/\delta$^38^ which contains all the information about the life history necessary to determine the long-term outcome in the mean field limit:

3 $$\widehat{p}=\{\begin{array}{ll}1-{{ {\mathcal R} }_{0}}^{-1}, & {\rm{if}}\,{ {\mathcal R} }_{0} > 1\\ 0, & {\rm{otherwise}}\end{array}$$

This approximation has its limitations which we see when comparing the critical extinction of the mean field limit (${\delta }_{c}=1$) with the CP (${\delta }_{c}\approx 0.6065$) for ${r}_{\ast }=1$. The diminished ${\delta }_{c}$ in the CP is due to crowding of populations which leads to some portion of colonization events to fall on already occupied patches. Spatial structure adds limitations to the efficacy of colonization, this is emphasized as we approach ${\delta }_{c}$.

### Ephemeral landscapes

Next, we consider a landscape where patch lifetime is ephemeral (*sensu* Keymer *et al*.^39^), therefore, aside from the landscape being occupied or vacant we add a third possible state, destroyed. This signifies a degradation in the local habitat thus leaving it unavailable for immediate colonization. All patches, regardless of state, are destroyed at rate $e$, if no mechanism for habitat renewal exists, the entire lattice converges to completely destroyed $\bar{s}=0$ where $\bar{s}$ stands for long term suitable habitat. Keymer *et al*. investigated the extinction threshold of the contact process on a dynamic landscape defined by global rates of habitat destruction ($e$) and renewal ($\lambda$) where they envisioned $\lambda$ as an ecosystem service independent of the metapopulation dynamics. Aside from the necessary conditions for the life-history of the population (i.e. ${ {\mathcal R} }_{0} > 1$), the extinction threshold is dependent on two aspects of the landscape;

(i). patch lifetime, $$\tau \equiv \frac{1}{e}$$
(ii). and long term suitable habitat, $\bar{s}=\frac{\lambda }{\lambda +e}$

If patch lifetime is shorter than some critical time span ($\tau  < {\tau }_{min}$), the dynamic corridors generated by habitat renewal are too short lived for populations to navigate through via colonization of vacant patches, likewise, below a minimum amount of suitable habitat ($\bar{s} < {\bar{s}}_{min}$), a spanning cluster of destroyed patches percolates the landscape leaving clusters of populations fragmented from suitable habitat^40^. In both cases the metapopulation enters the absorbing state (global extinction).

### Coupling niche construction to metapopulations in ephemeral landscapes

In order to connect metapopulation dynamics to the generation of the landscape, we introduce a niche constructor strategy (NC) (${\xi }_{t}(x)=1$) which, along with having the capacity to recruit local vacant sites (${\xi }_{t}(y)=\varnothing$) via immediate colonization, is also capable of converting local destroyed sites (${\xi }_{t}(y{\prime} )=-\,1$) to vacant and available for future colonization events. Thus, we are left with the following set of reactions (Fig. 1 for schematic):

$$\begin{array}{lll}{1}_{x} & \mathop{\longrightarrow }\limits^{\delta } & {\varnothing }_{x}\\ {\varnothing }_{x},{1}_{x} & \mathop{\longrightarrow }\limits^{e} & -\,{1}_{x}\\ -\,{1}_{x}+{1}_{y} & \mathop{\longrightarrow }\limits^{c/Z} & {\varnothing }_{x}+{1}_{y}\\ {\varnothing }_{x}+{1}_{y} & \mathop{\longrightarrow }\limits^{{(1-c)}^{\alpha }/{Z}_{\ast }} & {1}_{x}+{1}_{y},\end{array}$$

Notice that neighborhood sizes for colonization Z_*_ and construction *Z* are not necessarily the same. Furthermore, the capacity for construction $c$ comes at an energetic cost to colonization: ${(1-c)}^{\alpha }$ where the value of *α* determines the efficacy of niche construction.

Replacing the global ecosystem service rate $\lambda$ with one dependent on the state of local populations we can study the coupling between the metapopulation and landscape.

The model described above has 6 parameters; population extinction $\delta$, patch destruction $e$, patch construction $c$, construction efficacy $\alpha$ and the two neighborhood sizes for colonization Z_*_ and construction $Z$. The mean field approximation for this version of the model will be introduced next, followed by a comparison to the IPS. After presenting these results we make an addition to the model where we add a second strategy (exploiter, where $c=0$) which competes for colonizable space (scramble competition). We then discuss results in the context of landscape dynamics and competition.

### Mean field approximation

Here we consider the mean field approximation for the model with NC in an ephemeral landscape. Since global densities sum to unity we substitute for one of the states. For our purposes, we chose the global density of vacant patches ${p}_{\varnothing }=1-{p}_{+}-{p}_{-}$. Now we can write down the mean field approximation just considering the dynamics of destroyed ${p}_{-}$ and occupied patches ${p}_{+}$:

4 $${\dot{p}}_{-}=e(1-{p}_{-})-c{p}_{+}{p}_{-}$$

5 $${\dot{p}}_{+}={(1-c)}^{\alpha }{p}_{+}(1-{p}_{+}-{p}_{-})-{p}_{+}(\delta +e)$$

Besides the absorbing state ${\widehat{p}}_{-}=1$ there exists a steady state equilibrium ${\widehat{p}}_{+} > 0$. Although the exact solution is not particularly insightful, some observations are in order: we can determine the long-term suitable habitat

6 $$\bar{s}=1-{\widehat{p}}_{-}=\frac{c{\widehat{p}}_{+}}{c{\widehat{p}}_{+}+e}$$

Note, $c{\widehat{p}}_{+}$ can be defined as the total effort towards niche construction by the metapopulation. Substituting in the following Λ ≡ $c{\widehat{p}}_{+}$ we have returned to the expression for suitable habitat^39^ which is now coupled to the state of the metapopulation:

7 $$\bar{s}=\frac{\Lambda }{\Lambda +e}$$

For our model this expression is equivalent to the fundamental niche for the specific life history-landscape mapping in question and, like Levins expression for metapopulation occupancy, we can describe the abundance or realized niche as:

8 $${\widehat{p}}_{+}=\bar{s}-\frac{\tilde{\delta }}{{(1-c)}^{\alpha }}$$

where, $\tilde{\delta }=\delta +e$. In the case where $e=0$ and $c=0$ we return to the canonical equation for metapopulation occupancy (Eq. 2).

In the next section we compare the analytic results of the mean field with numerical simulations of the IPS. We use a von Neumann neighborhood for colonization and construction. Colonization neighborhood is fixed to the nearest neighborhood ($\parallel {Z}_{\ast }\parallel =4$). Unless specified otherwise, the construction neighborhood (||*Z*||) is identical. However, in order to study the transition from monopolized to exploited niche we will consider other interaction ranges for construction ($r$) in which case the construction neighborhood can be computed accordingly:

9 $$\parallel Z\parallel =2r(r+1)$$

We explored the 2D parameter space generated by the landscape dynamics $e$ and population strategy $c$ with respect to mean occupancy $\langle {p}_{+}\rangle$ through Monte Carlo methods. To study long-term behavior, the lattice was completely occupied to start all simulations.

## Results: Niche Construction Model

### Mean field approximation and interacting particle system comparison

**Figure 2:**
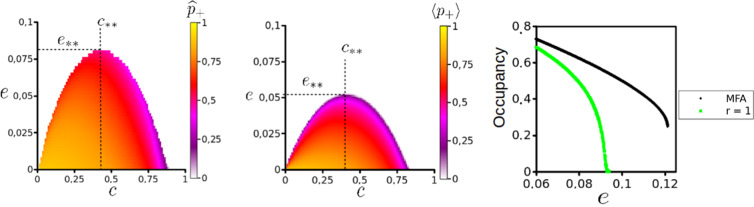
Long-term behavior of the NC Model. Results from the MFA (Left) and the IPS (Center) parameter spaces, with the life-history strategy adopted ($c$) along the x-axis and dynamics of the landscape ($e$) in the y-axis. For both $\delta =0.1$ and $\alpha =1$. For IPS, simulations ran for 5000 times steps, where, upon reaching the steady stateinvariant measure, a mean occupancy $\langle {p}_{+}\rangle$ was calculated from the following $250$ time steps. In total, 10,000 simulations were run by sweeping the parameter space $\{c,e\}$ creating a 100 × 100 matrix. Right: Divergence in the qualitative behavior of the niche construction model. For the MFA a discontinuous transition exists for the order parameter ${\widehat{p}}_{+}$, whereas the discontinuity disappears in the IPS for $r=1$ and we are left with a continuous transition, similar to the CP. Other parameters for MFA and IPS $\{\alpha ,\delta ,c\}=\{1,0,0.4\}$.

From Fig. 2, it is clear that the steady state equilibrium of the MFA overestimates the region of parameter space where the metapopulation can persist. Comparing the plots in Fig. 2 we see the curve $e(c)$ defines the population extinction threshold. MFA and IPS alike, the optimal trade-off between colonization and construction is landscape dependent. Tracing along the edge of the x-axis (where the landscape is almost static) we see the highest occupancy where little investment is made towards construction. This is due to the limited impact of destruction on the outcome of the metapopulation. As the rate of habitat destruction increases (i.e. as habitat lifetime decreases, $\tau \to 0$), strategies with conservative efforts towards construction (small $c$) cannotcan not persist and an increased investment towards the maintenance of the niche is necessary for metapopulation survival. Notably, the vertex of the extinction threshold curve (construction strategy c_**_ which persists in the broadest range of landscapes) is around c_**_ = 0.4 for both IPS and MFA, qualitatively similar to the conclusion made by Krakauer *et al*. for the single strategy system.

**Figure 3:**
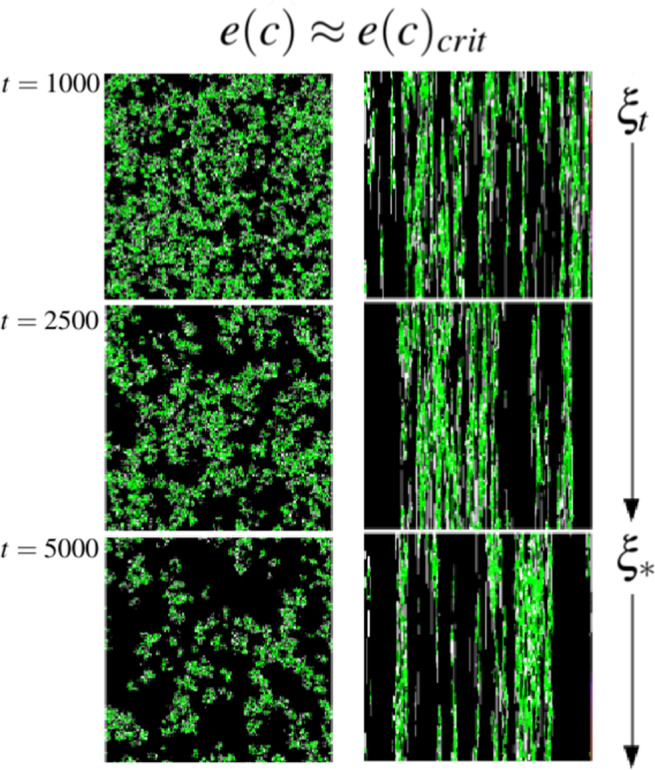
Left: Snapshots of the particle system at $t=1000,2500,5000$. Occupied (green), Vacant (white) & Destroyed (black) sites. Right: A 1D spatial transect of the particle system for 256 time steps. After a transient period (${\xi }_{t}$) the final snapshot/transect shows the system after it has reached the steady stateinvariant measure (${\xi }_{\ast }$). Parameter values used for this simulation were at the edge near the extinction threshold (See IPS in Fig. 2); $\{\alpha ,\delta ,c,e\}=\{1,0.1,0.25,0.045\}$.

Figure 3 shows a simulation where the metapopulation is able to persist in an ephemeral landscape even when confined to small clusters. These clusters are able to sustain themselves and create the corridors within the landscape (See 1D spatial transects on R.H.S of Fig. 3), thus escaping extinction. Although individual patches are fleeting, the metapopulation persists. It should be noted that near the extinction threshold, as shown in Fig. 3, the occupied patches always self-organize into these clusters for $r=1$. This landscape level spatial organization has been recognized as an early warning signal for arid ecosystems about to transition to a desert state^41^.

While the quantitative difference in the MFA and IPS extinction threshold is expected, the qualitative differences observed between MFA and IPS were not anticipated. Following the behavior of the deterministic MFA a sudden transition occurs at $e{(c)}_{crit}$, discontinuously jumping to extinction. Considering this result, one would expect the IPS to transition similarly (i.e. discontinuously) to the absorbing state. Instead we are left with a continuous phase transition, much like the one documented for the contact process. This observation suggests that our model falls in a larger group of mathematical models whose properties are universal and independent of the dynamical details, specifically, the Directed Percolation Universality Class^37^. To confirm this, one would need to perform finite size scaling analysis in order to derive the critical exponents of this model^42^.

**Figure 4:**
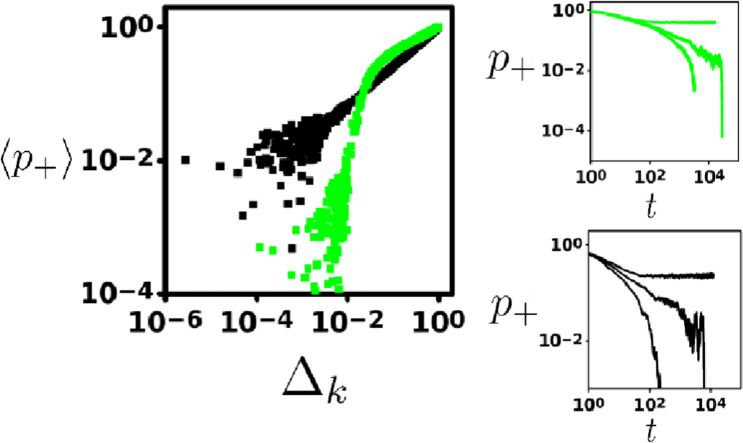
Left: Log-log plot showing critical behavior of the order parameter (long-term occupancy, $\langle {p}_{+}\rangle$) as we approach the critical point ${\delta }_{crit}$ and ${e}_{crit}$ for the contact process (black) and NC model (green), respectively. As these systems near the critical point (Δ_*k*_ ≡ $|k-{k}_{crit}|$ for $k=\delta ,e$), the order parameters display power law behavior with unique critical exponents (indicated by the unique slope for each). The critical exponent for the contact process determined from this slope (≈0.6116) holds up well against previous estimates^37^. Parameters for the niche construction model $\{\alpha ,\delta ,c\}=\{1,0,0.4\}$. Right: Dynamical behavior for the Niche Construction model (Top) and Contact process (Bottom) for $k < {k}_{crit}$ (subcritical), $k={k}_{crit}$ (critical) and $k > {k}_{crit}$ (supercritical), from top to bottom in each plot. Divergence of relaxation time to extinction at the critical point ($k={k}_{crit}$) is indicative of continuous phase transitions.

Typical of continuous phase transitions there exists a scaling regime; where the order parameter $\langle {p}_{+}\rangle$ behaves as a power law it approaches the critical value for extinction/habitat destruction (${\delta }_{c}$, ${e}_{c}$) in both CP and NC particle systems (L.H.S., Fig. 4). Furthermore, both display similar critical slowing-down divergence of the relaxation time as shown by 3 simulations run for values below, at and above the critical point. This is suggested by the linear decrease in population size shown on the log-log plots on the R.H.S. of Fig. 4 at the critical points for the NC (top) and CP (bottom) models. Due to finite size constraints, fluctuations in this linear decrease emerge as ${p}_{+}\to 0$.

### Effect of niche construction neighborhood on range expansion and resiliency

**Figure 5:**
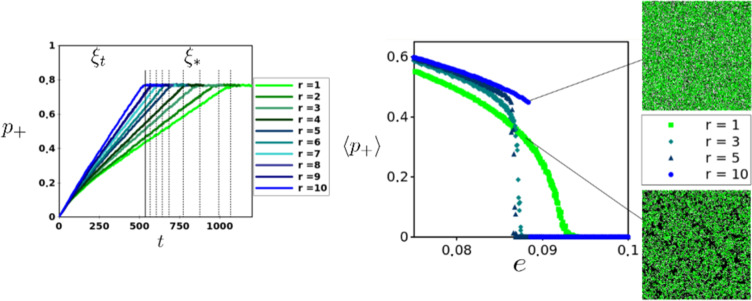
Left: Range expansion for niche constructor strategies with different *r*. After a transient period of expansion (${\xi }_{t}$) which decreased as *r* increased, identical $\langle {p}_{+}\rangle$ were reached for $e\ll {e}_{crit}$. The solid and consequent dotted vertical lines indicate the times (${\xi }_{\ast }$) when each strategy ($r=10,9,\ldots ,1$) reached $\langle {p}_{+}\rangle$. Simulations were run with the following parameter values $\{\alpha ,\delta ,c,e\}=\{1,0.1,0.4,0.01\}$. Right: Long-term behavior for different $r$ around ${e}_{crit}$. Parameters used for simulations were $\{\alpha ,\delta ,c\}=\{1,0,0.4\}$. Snapshots showing differences in spatial clustering taken from simulations at $e=0.088$ for $r=10$ and $r=1$.

In light of these striking discrepancies between spatial model and MFA prediction, we investigated the effect of construction range ($r$) on range expansion by the metapopulation starting from a single occupied site. During the initial expansion (${\xi }_{t}$) the metapopulation is both colonizing vacant sites and renewing destroyed sites for future colonization. The landscape quickly becomes divided into two distinct spatial regions, *i*) A pattern of occupied, vacant and destroyed sites which maintains its heterogeneity via the extinction ($\delta$), destruction ($e$) and construction ($c$) dynamics^43^ and which we refer to as a ‘mosaic’ *sensu*^44^ and *ii*) A homogeneous vacuum of destroyed sites where no local populations are nearby to combat the global destruction rate ($e$). While the exact configuration within the mosaic is constantly changing stochastically, a dynamical internal homeorhesis allows the metapopulation to not only persist, but expand as long as the landscape/population strategy mapping falls within the $e{(c)}_{crit}$ curve. Interestingly, at the boundary of these two spatial regions (mosaic and vacuum) we observe an edge effect: It is here, at the expansion front, where populations along the periphery of the mosaic can access the nearest sites of the vacuum, allowing them to convert destroyed sites into vacant sites, facilitating future expansion. As $r$ becomes larger we observe a steady increase in the rate of range expansion (therefore a shorter transient period, ${\xi }_{t}$) eventually leading to the same long-term average occupancy ${\widehat{p}}_{+}$ at which point the vacuum vanishes (Fig. 5). Intuitively, a larger $r$ means a greater portion of occupied patches are able to convert destroyed patches into vacant niche at the mosaic-vacuum interface. Furthermore, periphery populations are able to renew destroyed sites beyond adjacency, therefore widening the gap between mosaic and vacuum which in turn increases the chance for propagules to land on vacant sites. Interestingly, the spatial texture of the edge changes as niche construction neighborhood, $r$, increases. A gap emerges between mosaic and vacuum because metapopulation expansion is no longer tightly coupled to niche expansion like in the adjacent niche construction strategy ($r=1$). This subtle change at the mosaic-vacuum interface creates ecological opportunities which we address in the next section.

While this decoupling between the constructed niche and the local NC population (where $Z > {Z}_{\ast }$) does not come at an explicit additional cost since we consider the cost fixed for construction ${\mathrm{(1}-c)}^{\alpha }$, it has surprising implications for the resiliency of the metapopulation. As we approach $e{(c)}_{crit}$ from below, $\langle {p}_{+}\rangle$ begins to increase steadily with larger $r$. However, we observe a qualitative change in the behavior of the model at $e{(c)}_{crit}$ for $r > 1$; as $r$ increases the transition to the absorbing state becomes steeper, eventually displaying a discontinuity, as shown for $r=10$, and similar to the MFA. Additionally, the spatial clustering exhibited for $r=1$ near $e{(c)}_{crit}$ disappears. This last observation happens to be the key to understanding why the extinction threshold changes qualitatively as $r$ increases. To do so, it is useful to return to the mosaic-vacuum spatial paradigm. Now consider starting with a fully occupied lattice ${p}_{+}=1$. For small $r$ as $e\to {e}_{crit}$ the metapopulation structure becomes fragmented, but clusters continue to persist (Fig. 3). These clusters of occupied patches (mosaic) drift through the sea of uninhabitable patches (vacuum) occasionally fusing with other clusters and occasionally going extinct. Since this drift depends on their ability to renew the niche at the expansion front, they benefit from the highly condensed internal structure of the mosaic and the tight coupling with recent niche construction events. Internally, they maintain homeorhesis while capitalizing most effectively on renewed niche at the edge because any renewal events are likely to be adjacent to at least one viable population. If $e={e}_{crit}$ then the relaxation time to extinction behaves as a power law as expected for continuous absorbing state phase transitions^37^. As $r$ increases, clustering is lost for the same reason it aids in range expansion; instead of the decoupling between vacant niche and mosaic being generated at the expansion front of the metapopulation it exists over the entire landscape. Therefore, when $e={e}_{crit}$, given enough time an area of the landscape will return to the vacuum. In contrast to the sub-critical regime, the vacuum now expands and the mosaic compresses, thus fragmenting the metapopulation. Since the niche construction neighborhood is large, no clustering occurs and the system experiences a rapid relaxation time to the absorbing state. While a larger neighborhood of niche construction increases range expansion it also makes the metapopulation more vulnerable to global extinction by changing the coupling between niche and population. At the larger scale this is observed by the disappearance of spatial clustering.

## Addition to the Model

### Competitive scramble for ephemeral patches

To further explore the colonization-niche construction trade-off, we introduce an additional strategy to see how competition between two life-history strategies affects the long-term outcome of metacommunity persistence in this ephemeral landscape.

**Figure 6:**
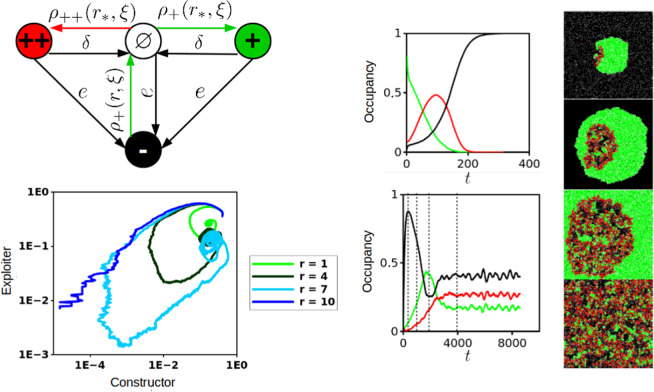
Top Left: Updated schematic of model with NC (green) and exploiter (red) competing for space. Bottom Left: Trajectories of the constructor-exploiter phase space for different $r$ values. Larger values of $r$ increase the likelihood of ecological suicide during the transient period. Oscillations are caused by range expansion/exploitation of the NC/ES populations. If the NC metapopulation can escape the first wave of exploitation from the exploiter strategy then coexistence is likely. Center: Population dynamics for global (top) and $r=1$ (bottom) niche construction neighborhood following niche constructor (green) contact process (red) strategies along with destroyed habitat (black) in the coexistence regime: $\{\alpha ,\delta ,c,e\}=\{1,0.1,0.4,0.01\}$. Right: Four snapshots taken at $t=250,1000,2000,4000$ from top to bottom, temporal locations of snapshots are indicated with vertical dashed lines. Frame 1 shows the exploiter population being fragmented from vacant sites due to habitat destruction, but rescued by the NC population expanding via the construction of viable habitat. Once the exploiter population establishes itself within the confines of the surrounding NC particles (frame 2 and 3) they can begin exploiting this renewed habitat and freshly vacated sites (generated by $c$ and $\delta$, respectively) due to their superior colonization rate. Eventually, the system equilibrates, although small fluctuations continue due to the local oscillations and the finite size of the landscape, see^63^ for a discussion on this with respect to lattice models.

We introduce the basic contact process (See *Section 2*.*1*.) into the model as an exploiter strategy. Since the exploiter does not partition effort to construction it has a higher ${ {\mathcal R} }_{0}$ than NC. Given a static landscape without destruction, the larger ${ {\mathcal R} }_{0}$ will always push the smaller ${ {\mathcal R} }_{0}$ strategy to eventual extinction. This example of the competitive exclusion principle^45^ has been proven rigorously^46^ in the context of competing contact processes. However, any non-zero destruction rate requires some investment in construction in order to persist in the landscape. Therefore, we would expect the exploiter to behave as a fugitive species^47^, more capable of colonizing vacant niche, but unable to maintain a spatially connected metapopulation through time. For this model we do not assume any competitive hierarchy between the two strategies. An updated schematic of the complete model is provided (Fig. 6).

## Results: Scramble Competition Model

We first compare the dynamics of the model when construction is completely uncoupled to the NC (i.e. $\parallel Z\parallel = {\mathcal L}$), versus when it is tightly coupled to the NC strategy ($\parallel Z\parallel =\parallel {Z}_{\ast }\parallel$) in Fig. 6. We see that for the global case, exploiter out-competes the NC strategy globally, leading to its extinction. Once this occurs, nothing prevents the lattice from becoming completely destroyed. For future reference, we refer to this outcome as ‘ecological suicide’ since we do not consider evolving populations. In the tightly coupled case, where $r=1$, we see a drastically different outcome in which the two strategies coexist.

Coexistence is possible because NC has the advantage in adjacency to the constructed niche, while exploiter has a colonization advantage (larger ${ {\mathcal R} }_{0}$), this adjacency advantage diminishes for increased $r$. In the landscape, exploiter populations chase clusters of NC leading to the oscillations observed in Fig. 6. With increased $r$, the fluctuations of the metacommunity occupancy increase in intensity because exploiters more effectively exploit renewed niche generated by NC populations both at the mosaic-vacuum interface and within the mosaic. While increased $r$ leads to a faster rate of range expansion for the constructor in isolation, this benefit is lost when in scramble competition against exploiters.

If NC populations become surrounded by the exploiter they are likely to go extinct, unless a gap in the perimeter is created by either extinction or destruction events. Therefore, the NC strategy is only able to persist if they can access the interface with the vacuum.

**Figure 7:**
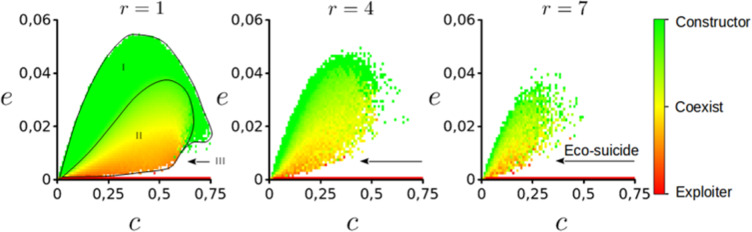
Parameter spaces {*c*, *e*} for the model with scramble competition given three different niche construction neighborhoods, *r* = 1, 4 & 7 from left to right. Relative occupancy is measured as $\frac{\langle {p}_{+}\rangle }{\langle {p}_{+}\rangle +\langle {p}_{++}\rangle }\in [0,1]$. Additional parameter values $\{\alpha ,\delta \}=\{1,0.1\}$.

Along with the previously observed instances where the landscape - population strategy mapping pushes the NC metapopulation to extinction (the trivial case) in the single strategy model, there exist 4 phases of the parameter space (Fig. 7), described as follows:

(i). High disturbance regime; Where constructor metapopulations can successfully monopolize the constructed niche due to the short life-span $\tau$ of patches leading to exploiter fragmentation and extinction. By escaping exploitation, the NC strategy is able to persist at the same global occupancy ${\widehat{p}}_{+}$ as in the single strategy scenario.
(ii). Intermediate disturbance regime; Coexistence between NC and exploiter. Here a dynamic equilibrium is achieved due to a balance in the construction-colonization trade-off. In this regime exploiters are faster at colonizing constructed niche without completely exploiting the constructor population, which maintains itself through range expansion. Within this regime we see an overall decrease in patch occupancy ($\langle p\rangle$) but increase in total diversity reminiscent of the intermediate disturbance hypothesis^48^.
(iii). Low Disturbance regime causing ecological suicide; Exploiter forces constructor to extinction by effectively over-exploiting the constructed niche to the point of enclosing small clusters of constructor populations. If the constructor strategy cannot escape this confinement, they eventually go globally extinct resulting in the subsequent collapse of the niche and remaining exploiter metapopulation.

And, finally the iv) No disturbance regime, where competitive exclusion pushes the constructor to extinction collapsing the exploiter metapopulation. This regime is limited to the red horizontal line at the bottom of the parameter space where $e=0$.

From these results we are interested in the transitions from one regime to the next when considering $e$ and $r$. One striking discovery is the onset of ecological suicide from above $ii\to iii$ and below $iv\to iii$ which is enhanced with an increased effort towards niche construction ($c\to 1$), and expands in parameter space with increasing $r$. In contrast and counter-intuitive to expectations, we see an increase in constructor strategy occupancy as a response to increasing ephemerality of patches as we transition from $ii\to i$. This transition becomes noisier as $r$ increases.

Interestingly, for $r > 1$, stochastic events on the lattice can determine the long-term outcome of the metacommunity. When a simulation starts, exploiter patches quickly colonize territory around NC patches which self organize into clusters. For $c\gg 0$ clusters of NC become encircled by exploiter patches, which eventually choke-out NC populations leading to ecological suicide. If however, they are able to slip past exploiter patches and reach the vacuum, they can continue to expand their range, at which point exploiter patches connected by vacancy will continue to follow their expansion. Alternatively, if exploiter patches become separated entirely from the NC metapopulation, exploiter populations will stay fragmented from vacant niche and eventually be vanquished by this self-inflicted fragmentation and the NC metapopulation will return to the long-term behavior of phase i). It is these mechanisms which lead to the noisy behavior of the model at the edge of the extinction threshold, and consequently, this noisiness is enhanced as the difference in size between colonization and construction neighborhoods increases from a relatively monopolized good (${Z}_{\ast }=Z$) tightly coupled to the NS, to a public good (${Z}_{\ast } < Z$), easily exploited by the ES.

## Discussion

Our goal here has been to implement a spatial extension of the constructed niche as defined by Krakauer *et al*.^16^, through the interacting particle system framework, in order to determine if a spatial perspective would reveal new and interesting insights into the ecological consequences of niche construction in ephemeral landscapes. While some observations made by Krakauer *et al*. were reconfirmed here in the spatial model, for example the optimal trade-off between construction and colonization in the single strategy system, new significant findings were also uncovered; for instance, in cases where niche construction is localized ($r=1$) leading to the emergence of clustering. This clustering in turn allowed the metapopulation to maintain itself where larger $r$ could not. This outcome was reminiscent of the bi-stability predicted by the MFA. In contrast to these mean field predictions, no such behavior was observed for the spatial model when $r$ was limited to adjacent sites. Monte Carlo simulations revealed a continuous phase transition which was steeper than, but qualitatively similar to the contact process. As we increased $r$ for the IPS this transition became more drastic, until it eventually vanished and the discontinuity predicted in the MFA was recovered.

Continuing our study of the single strategy model, we investigated the impact of $r$ on range expansion. As expected, increased niche construction neighborhoods led to an increased rate of range expansion. Therefore, before implementing a second strategy into the model an unanticipated trade-off existed for the niche construction strategy between the rate of range expansion and susceptibility of the metapopulation to critical transitions.

Next, we studied the two strategy model, where the regular contact process behaved as an exploiter strategy to the niche constructor. As expected, increased niche construction neighborhood allowed for greater exploitation. For phases of the parameter space, the system experienced what we referred to as ecological suicide, where exploiter pushed NC to extinction, thus sowing the seeds to their own demise. Unexpectedly, extinction due to ecological suicide was not as clearly defined in our parameter space as the single strategy phase transitions. For example, unlike in the single strategy model where we located ${e}_{crit}$ with extensive Monte Carlo simulations, we are unable to define critical neighborhood size where ecological suicide begins. This is possibly due to the stochastic nature of its mechanisms; wherein, clusters of NC sites capable of escaping local extinction via scramble competition are freed from the exploiter strategy allowing them to reach equilibrium values equivalent to the single strategy model. For $r=1$, ecological suicide is most probable when $e$ is small and $c$ large because the NC reproductive ratio (${ {\mathcal R} }_{0}$) is compromised by a high investment in niche construction. This allows the exploiter to quickly out-compete and surround NC before being fragmented by patches of destruction. As $r$ increases the speed with which the exploiter is able to surround NC increases. The expanding area of the parameter space where ecological suicide occurs as $r$ increases exemplifies the tragedy of the commons described by Krakauer *et al*.^16^.

This result emphasizes the importance of spatial structure on the long-term and global outcome of niche constructor/exploiter metacommunity dynamics. Notably, how a few stochastic events changing the connectivity of patches between populations of niche constructing and exploiting strategies lead to drastically different outcomes. Specifically, the initial conditions of these founder populations (clusters of occupied sites surrounded by destroyed sites) can exhibit both stochastic (dominated by random chance events) and deterministic phases (predicted by the proportion of NC vs. CP sites in a cluster)^49,50^. In this case, the transition between these two phases is determined by $r$.

Experimental results studying the spatial dynamics of a pair of engineered cross-feeding (niche constructor) *S*. *cerevisiae* and one non-reciprocating (exploiter) strategy also observed the spatial clustering in our models results of Fig. 6 ^51^. The authors emphasize the self-organizational clustering as an effective assortment mechanism for individual cells to gain cooperative partners and exclude defectors.

Furthermore, clinical research following the colonization dynamics of *P*. *aeruginosa* in the lungs of mechanically ventilated patients show similar dynamics as our constructor-exploiter model^52^. The *P*. *aeruginosa* populations of patients monitored over a period of 3 weeks showed a rapid decline in quorum sensing diversity, emphasized by an increase of the lasR mutant (lasR) which likely evolved from the wild-type (wt) strain. The lasR mutant is considered an exploiter as it does not respond to the quorum signal but is able to benefit from the catabolic enzymes produced by wild-type strains and in this way creating a selective advantage^53^. The authors note that this advantage was only found whilst in the presence of the wild-type leading to fluctuations in occupancy between the exploiter (lasR) and constructor (wt) and reminiscent of the temporal dynamics of our model (see Fig. 6) and the long-term equilibrium of the coexistence regime (ii). In addition, in patients where lasR did not successfully invade the population (i.e. only wt strategy), ventilator associated pneumonia occurred significantly faster suggesting, as our model does, that the release of exploitation increases the carrying capacity of the metapopulation in regime i.

Inspired by the earlier studies documenting this tragedy of the commons in *P*.*aeruginosa*, Jin *et al*. laid out how the production and excretion of these exploitable siderophores is modified in the presence of environmental stress ($e$ in our model and $[]$ tobramycin here)^54^. Using *in vivo* observational experiments, they discovered wt cells are capable of tuning the efflux pump responsible for the excretion of pyoverdine (siderophore) based off the stress level of the environment, in this way being able to monopolize the constructed niche in stressful environments. Using a well-mixed competition experiment, they were able to show that while moderately stressful environments (small $e$) reproduced the observations of^26^ i.e. exploitation by mutant strain leading to wt extinction, more stressful environments (large $e$) counter-intuitively led to wt dominance characterized by this ‘conditional privatization’ (See Fig. 4 in^54^). While these experimental observations verify the qualitative behavior of our model results defined by regimes i-iv, yet verified is the effect $r$ has on the shape of these regimes as in Fig. 7. We think range expansion experiments would be an ideal experimental set-up to study the role of space in constructor-exploiter dynamics^55^.

While an extensive literature exists for studying spatial games in ecology and evolution^56–58^, much of this is built on a strategy-strategy payoff matrix. Theoretical advances aimed at considering environmentally mediated resources, in our case the constructed niche, are still in their infancy^59,60^.

## Conclusion

We envision the trade-off between colonization and construction to be a generalized predicament faced by populations and whose solutions to this dilemma mark the most significant transitions in the evolution of life on earth^61^. Of particular interest is the origins of multicellularity, which was most likely achieved through the division of labor between maintenance (construction) and replication (colonization)^20,62^. Here we developed a ‘toy ecology’ within the interacting particle system framework^56^ which we believe reproduces some of the conceptual dilemmas faced at this transition from population to individual. Furthermore, these findings have implications for the study of ecosystems stability and susceptibility to critical transitions. Understanding which real world ecosystems are likely to behave as continuous vs. discontinuous phase transitions is crucial for their conservation and management.
